# Pediatric vertebral artery dissection and ischemic stroke following chiropractic manipulation

**DOI:** 10.1007/s00381-026-07229-5

**Published:** 2026-03-31

**Authors:** Jonathan C. Arnold, Andrew M. Brumett, Kyle J. Ortiz Rodriguez, Raj Swaroop Lavadi, Rabia Qaiser

**Affiliations:** 1 Department of Neurological Surgery, Indiana University School of Medicine, 355 West 16th Street, Goodman Hall 5100, Indianapolis, IN 46202 USA; 2 Department of Neurological Surgery, Riley’s Children’s Hospital, Indianapolis, IN USA

**Keywords:** Chiropractor, Cervical manipulation, Vertebral artery dissection, Stroke, Pediatric

## Abstract

Pediatric vertebral artery dissection (VAD) following chiropractic cervical manipulation (CCM) is a rare phenomenon. As chiropractic care of pediatric populations increases internationally, it is imperative to increase awareness of this cause of VAD. The patient encountered in our institution was a 20-month-old male who presented nonspecifically with acute onset of lethargy, vomiting, cyanosis, and respiratory distress. Cerebrovascular imaging revealed a luminal irregularity in the V4 segment of the right vertebral artery, consistent with dissection. The patient’s guardian later provided history of taking the child for cervical chiropractic corrections immediately prior to the patient’s presentation to the emergency department. The patient was managed non operatively and was later discharged without neurological deficit. Non-specific presentations were also noted in the other two cases described in the literature. Appropriate diagnosis of pediatric VAD requires increased surveillance in response to a thorough history and an acknowledgment of the plethora of possible patient presentations and etiologies.

## Introduction

Vertebral artery dissection (VAD) following chiropractic cervical manipulation (CCM), although well-documented in adults, is less reported in children [1]. Unlike adults, where neck pain is common, children often present with nonspecific symptoms such as ataxia, headache, vomiting, and/or paresis/paralysis of one or more extremities [1, 2]. Considering the increased utilization of chiropractors in pediatrics, [3] an adequate description of the risks of manipulations is paramount. The following report presents our experience with this etiology. Ethics review was not required, and patient consent was obtained from legal guardians.

## Case presentation

**Fig. 1:**
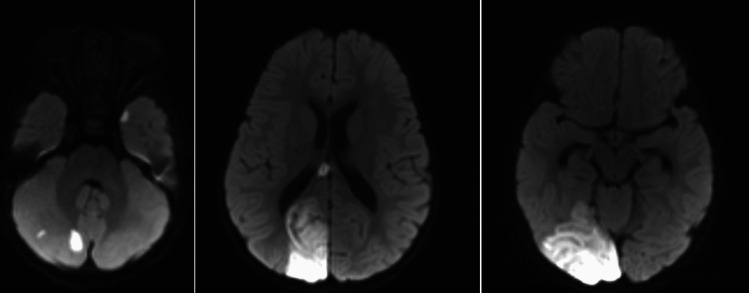
Diffusion-weighted images showing multifocal infarcts of the right posterior occipital lobe and right cerebellum

**Fig. 2:**
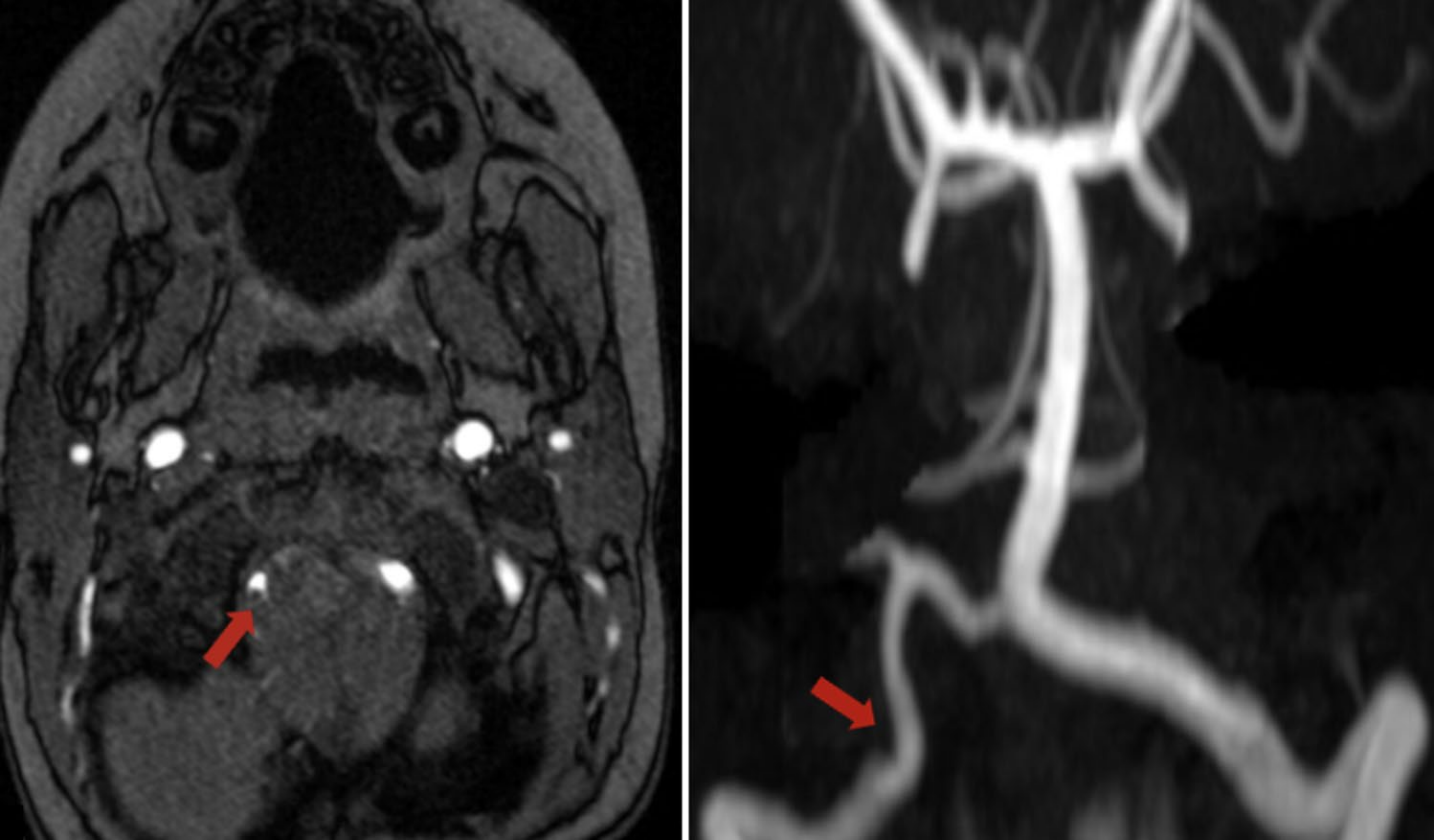
MRA Brain demonstrating luminal irregularity in the V4 segment of the right vertebral artery consistent with dissection

**Fig. 3:**
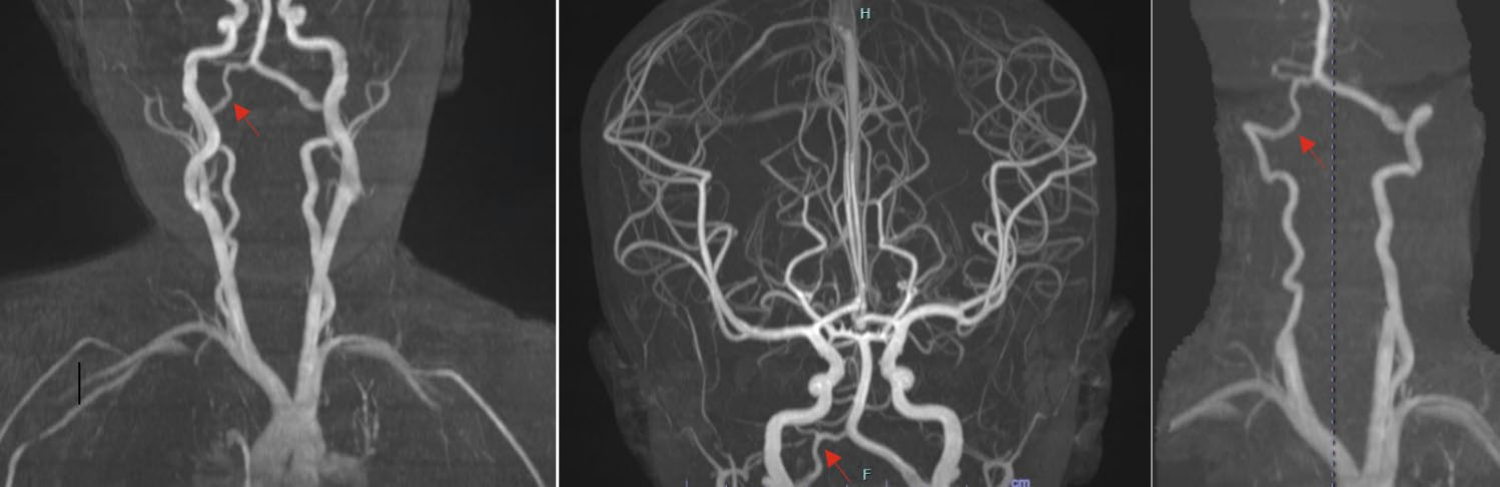
MRAs of the brain and neck demonstrated a hypoplastic right vertebral artery (arrow)

A 20-month-old male with history of poor weight gain and asthma attacks presented to the emergency room with acute onset of lethargy, vomiting, cyanosis, and respiratory distress. On the day of presentation, the parents took the patient to visit a gastroenterologist and a chiropractor to address his poor weight gain. The chiropractor allegedly performed CCM, which the patient initially tolerated well. No history of head trauma was reported by the patient’s guardian. On examination, the patient opened eyes to light touch, was grunting, and moving all extremities symmetrically and against gravity when stimulated. The patient also exhibited lethargy, vomiting, cyanosis, and respiratory distress. The patient’s pupils were equal and reactive. Vitals were as follows: HR 194 bpm, RR 46/min, Temp 37.1C, O_2_ saturation 88% on room air, and a systolic blood pressure of 66 mmHg. The patient had an elevated white blood cell count and was acidotic. Head CT showed sulcal effacement, cisternal crowding, and low-lying cerebellar tonsils, likely attributed to vasogenic edema. Non-contrast MRI demonstrated diffusion restriction in the right posterior cerebral artery territory and multifocal infarcts in the right cerebellum (Fig. 1). MRAs of the brain and neck demonstrated luminal irregularity in the V4 segment of the right vertebral artery consistent with dissection (Fig. 2), along with a hypoplastic right vertebral artery (Fig. 3). Infectious workup was positive for rhinovirus and enterovirus, and although lumbar puncture was delayed due to tonsillar descent, there was no CNS infection. Intubation was performed due to respiratory distress and managed with fluids, vasopressors, antimicrobials, and high-flow oxygen. The patient was extubated four days after presentation, and pressors were discontinued upon achievement of hemodynamic stability. A few days after extubation, the patient was ambulating and able to interact with objects and caretakers. Aspirin therapy was initiated and continued after discharge. The patient was followed with annual appointments and imaging. At two-year follow-up, CTA demonstrated an asymmetrically small right vertebral artery, accompanied by encephalomalacia of the right posterior occipital lobe. MRA demonstrated diffuse narrowing of the V4 segment of the right vertebral artery, albeit less pronounced than prior MRAs. Aspirin was discontinued by an outside following team due to stability of imaging findings. The parents were advised to avoid contact sports to avoid trauma and recurrent stroke.

Two other cases of pediatric VAD following CCM garnered from a literature search and their outcomes and treatment have been presented in Table 1 [4, 5].

**Table 1:** Details of prior cases of pediatric vertebral artery dissection following chiropractic cervical manipulation

Case	Age/Sex	CCM Indication	Symptoms	Imaging	Management	Outcome
Deputy 2014^4^	6-year-old male	Cervical manipulation for sinus infection 6 months prior to presentation	Gradual left-upper extremity paresthesia accompanied by left-upper extremity weakness	Spine MRI: “Snake eyes” infarctions of the bilateral ventral horns from C5-T1	Prednisone taper before visit, no VAD-specific treatment after clinical encounter	Persistent left arm motor weakness
Cochran, et al. 2020^5^	2-month-old male	Cervical manipulation since birth for torticollis	Seizure, lethargy, episodic limb movements	CT: hypodensities in bilateral PCA territories + MRI: bilateral PCA territory infarcts + MRA: V2 dissection	Aspirin, phenobarbital	Persistent epileptiform discharge, mild horizontal tracking issues

## Discussion

### Trends in cervical chiropractic care

Worldwide, chiropractic care has recently increased in popularity in both children and adults. Although chiropractic care is often perceived as low risk, it can carry serious adverse effects. A study that analyzed the 2012 National Health Interview Survey demonstrated that the lifetime usage of chiropractors by US adults was 24%, accompanied by an increased utilization of chiropractors from 2002–2012 [6]. This increased usage also applies to infants, with children aged 0–4 years constituting the largest pediatric demographic seen by chiropractors [7]. In parallel, a meta-analysis found that chiropractic spinal adjustments are often used in infants for the treatment of colic, torticollis, and plagiocephaly despite limited evidence of efficacy [8].

Although there is an increased usage of CCM in pediatrics, the efficacy of this treatment is not supported by evidence and there are no agreed-upon clinical guidelines for this intervention in pediatric patients [9]. Minor adverse outcomes following CCM are reported more often than severe complications such as VAD, but severe CCM complications are likely underreported in the literature [8, 9].

### Pediatric VAD

The vertebral arteries course cranially through the transverse foramina before entering the cranial cavity as the intradural V4 segment to form the basilar artery. VAD occurs after an intramural hematoma causes vascular occlusion secondary to an intimal tear [1, 2, 10]. In parallel to the increased usage of chiropractors in children, pediatric patients demonstrate increased risk to VAD relative to adults due to their unique anatomical features, including increased ligamentous laxity, a greater head-to-body ratio, and immature bony anatomy at the craniocervical junction. These characteristics increase the risk of pediatric VAD following minor trauma or forced neck movement, particularly at the V3 segment where rotational forces are concentrated. Children are also more likely than adults to possess osseous variants and congenital abnormalities, which can increase the risk of dynamic arterial compression and dissection during rapid movement [1, 11–13]. The inability of preverbal children to communicate their symptoms, in addition to non-specific pediatric VAD symptoms such as combinations of ataxia, headache, vomiting, and neck pain, complicates the diagnosis of pediatric VAD [1, 12]. Cerebrovascular imaging is indicated in children with posterior circulation stroke symptoms associated with neck pain, headache, or recent cervical trauma, and CTA or MRA of the neck are preferred [11, 12]. Common radiographic findings in pediatric VAD include luminal irregularity, pseudoaneurysm, intramural hematoma, and scattered posterior circulation infarcts [1, 2, 11]. In our case, MRA demonstrated luminal irregularity of the V4 segment associated with multiple posterior circulation infarcts on DWI, suggesting dissection. These findings suggest that the clinician should exercise a high index of suspicion for VAD when there is a history of CCM and should consider cerebrovascular imaging to rule out iatrogenic VAD [10].

## Conclusion

There is an increasing utilization of chiropractors among the pediatric population. In a pediatric patient with nonspecific symptoms, VAD should be considered as a differential diagnosis when there is a history of CCM.

## Data Availability

No datasets were generated or analysed during the current study.
